# Route knowledge and configural knowledge in typical and atypical development: a comparison of sparse and rich environments

**DOI:** 10.1186/s11689-015-9133-6

**Published:** 2015-12-15

**Authors:** Emily K. Farran, Harry R. M. Purser, Yannick Courbois, Marine Ballé, Pascal Sockeel, Daniel Mellier, Mark Blades

**Affiliations:** Department of Psychology and Human Development, UCL Institute of Education, University College London, 25 Woburn Square, London, WC1H 0AA UK; School of Psychology, University of Nottingham, Nottingham, UK; Laboratoire PSITEC (EA 4072), Université de Lille, Villeneuve d’Ascq, France; Laboratoire PSY-NCA (EA 4306), Université de Rouen, Rouen, France; Department of Psychology, University of Sheffield, Sheffield, UK

**Keywords:** Navigation, Spatial cognition, Williams syndrome, Down syndrome, Development

## Abstract

**Background:**

Individuals with Down syndrome (DS) and individuals with Williams syndrome (WS) have poor navigation skills, which impact their potential to become independent. Two aspects of navigation were investigated in these groups, using virtual environments (VE): route knowledge (the ability to learn the way from A to B by following a fixed sequence of turns) and configural knowledge (knowledge of the spatial relationships between places within an environment).

**Methods:**

Typically developing (TD) children aged 5 to 11 years (*N* = 93), individuals with DS (*N* = 29) and individuals with WS (*N* = 20) were presented with a sparse and a rich VE grid maze. Within each maze, participants were asked to learn a route from A to B and a route from A to C before being asked to find a novel shortcut from B to C.

**Results:**

Performance was broadly similar across sparse and rich mazes. The majority of participants were able to learn novel routes, with poorest performance in the DS group, but the ability to find a shortcut, our measure of configural knowledge, was limited for all three groups. That is, 59 % TD participants successfully found a shortcut, compared to 10 % participants with DS and 35 % participants with WS. Differences in the underlying mechanisms associated with route knowledge and configural knowledge and in the developmental trajectories of performance across groups were observed. Only the TD participants walked a shorter distance in the last shortcut trial compared to the first, indicative of increased configural knowledge across trials. The DS group often used an alternative strategy to get from B to C, summing the two taught routes together.

**Conclusions:**

Our findings demonstrate impaired configural knowledge in DS and in WS, with the strongest deficit in DS. This suggests that these groups rely on a rigid route knowledge based method for navigating and as a result are likely to get lost easily. Route knowledge was also impaired in both DS and WS groups and was related to different underlying processes across all three groups. These are discussed with reference to limitations in attention and/or visuo-spatial processing in the atypical groups.

## Background

Our ability to navigate successfully in large-scale space is crucial to everyday living [41]. Developmentally, we initially learn the way from A to B by following a fixed sequence of turns, using landmarks as reference points, a strategy referred to as the use of route knowledge [42]. Whilst this strategy is effective, it has limitations because it is inflexible to deviations from the known route. It is because young children rely on route knowledge that they easily get lost and find it difficult to re-orient themselves if they are forced to take a detour from their usual route.

A more flexible stage of route learning involves knowledge of the spatial relationships between places within an environment. This is referred to as configural knowledge or the ability to hold a ‘cognitive map’. Developmentally, children become gradually more able to use the configuration of the environment as a strategy to successfully find their way around (i.e. they develop a cognitive map) between 5 and 10 years of age [6]. This configural knowledge of the environment enables us to find shortcuts or alternative routes between two known places and to re-orient and find our way back if we get lost.

Route knowledge and configural knowledge are qualitatively different wayfinding strategies [31]. This is true at both the behavioural level and the neural level. That is, in adults, knowledge related to walking a fixed route activates the caudate nucleus, whilst knowledge related to the configural structure of an environment activates the hippocampus [22]. In the current study, we were interested not only in route learning abilities in typical children but also in two neurodevelopmental disorder groups, Down syndrome (DS) and Williams syndrome (WS). Individuals with DS and with WS have different uneven cognitive profiles, but both have atypicalities in the structure and function of the hippocampus (DS: [35]; WS: [29]). This suggests that configural knowledge is likely to be impaired in both DS and WS, but that performance will be associated with differing underlying mechanisms. Thus, the comparison of these two groups, along with typical development, offers insight into the mechanisms that act as limiting factors to the development of configural knowledge, either through impairment or immaturity. Furthermore, comparison between two different neurodevelopmental disorders will enable differentiation between syndrome-specific impairments and those associated with having more general learning difficulties. The aim of the current study was to investigate the development of both route knowledge and configural knowledge in these groups by asking participants to learn novel routes (route knowledge) and to find the shortest route between two known places in a virtual environment (VE) (configural knowledge). We also included a battery of attention tasks (discussed below), as mechanisms that we predicted would be associated with navigation performance.

Williams syndrome (WS) is a rare genetic disorder with a prevalence of between approximately 1 in 7500 [44] and 1 in 20,000 [32]. Individuals with WS present with moderate learning difficulties and a cognitive profile in which visuo-spatial abilities are weaker than verbal abilities [20]. Recent studies have explored route learning in both real-world and virtual environments in WS. This has demonstrated the ability to develop route knowledge of both real and virtual environments in this group [16–18, 36]. However, we know very little about large-scale configural knowledge in WS. In Farran et al. [16], the experimenter led participants with WS around a 1-km route and then asked the participant to lead the experimenter. As a measure of large-scale route knowledge, participants received a score out of 20 for the number of correct turns made. As a measure of large-scale configural knowledge, participants were asked to stop at four locations along the route and to point in the direction of three landmarks on the route that were not visible at that point in time. As stated above, participants developed route knowledge, i.e. they were able to learn the majority of turns along the route, but they had no understanding of the configural structure of the environment. However, as Farran et al. [16] report the same pattern of findings in a group of individuals with moderate learning difficulties (MLD) matched to the WS group by non-verbal ability, it does not appear to be syndrome specific. It is possible that the results do not represent a true failure to understand the configural structure in WS and MLD groups, but reflected a lack of opportunity to become familiar with the route because they were only given two opportunities to retrace the route. This latter consideration is countered by Broadbent et al. [1], who used a virtual environment cross-maze (a square maze with four radial arms). After extensive experience of the maze, participants were asked to walk the shortest route to one of four exits. Whilst typically developing 5- to 10-year-olds showed developmental progression from an egocentric route-based strategy to a configural strategy, the WS group showed atypical performance, often walking inefficient routes to reach the required exit. Furthermore, when asked to indicate which of the six maps represented the maze, they demonstrated a weak cognitive representation of the layout of the environment, also suggestive of poor large-scale configural knowledge. Individuals with WS are unable to use the geometry of a room to orient themselves in a re-orientation task, but can use a single landmark as a directional cue [26], which further supports the notion of poor configural knowledge in this group.

Studies that have investigated large-scale visual search in WS tap into different but overlapping skills compared to the skills measured by route learning studies. Both investigate configural information in WS, but visual search tasks use an open environment in which all landmarks are simultaneously visible. This makes the configural structure of the environment directly visible. In route learning tasks, participants walk along paths and most landmarks are only visible from the path on which they feature, with a few distant landmarks visible from multiple places within the environment. Thus, the configural structure is not directly visible. Three studies have explored large-scale visual search in WS, all of which report impairments which can, at least in part, be accounted for by impaired understanding of the configural structure of landmarks within the search array, despite the landmarks being simultaneously visible [21, 27, 43].

Evidence from small-scale tasks also suggests poor configural knowledge in WS. Nardini et al. [33] demonstrated that individuals with WS were unable to use the spatial configuration of landmarks to find a target. Broadbent et al. [5] and Bernardino et al. [2] demonstrated impaired performance in WS on three-dimensional perspective taking tasks. Success on such tasks requires an understanding of the spatial relationships among the objects in the array. More generally, individuals with WS display a local level bias (and thus reduced attention to global and configural information) on some small-scale visuo-spatial tasks such as block construction and drawing tasks (see [19]) and during face processing [24]. However, note that developmental studies do not consistently show a direct mapping between small-scale spatial skills and spatial knowledge on large-scale tasks (see [37]), and so we cannot assume that these deficits in WS are associated with the evidence above which suggests impaired configural knowledge on large-scale tasks in this group.

Down syndrome, also a genetic disorder, has a prevalence of between 1 and 3 per 1000 live births [11]. The level of IQ in DS broadly compares to that in WS, but the cognitive profile reflects weaker verbal than visuo-spatial abilities. The visuo-spatial cognitive profile observed in DS has been contrasted to that observed in WS; evidence from block construction and drawing tasks demonstrate a global level bias in DS (in contrast to the local level bias observed in WS) (e.g. [1]). However, the evidence below regarding large-scale spatial competence in DS does not suggest that this attentional bias towards global or configural information pervades to large-scale space. This is not surprising, given that small-scale local and global processing relate to processing styles, where as large-scale configural knowledge reflects a developmental endpoint, i.e. adult-level navigational ability which develops with cognitive maturity and experience (cf. [42]).

There have been four studies that have assessed navigation in DS. One of the studies discussed above also included a DS group [36]. Purser et al. [36] tested route knowledge, but did not test configural knowledge. They showed that individuals with DS could learn a six-turn route, but route knowledge in DS was much poorer than the WS group. Similarly, Davis et al. [10] reported that individuals with DS committed more errors in a route learning task than individuals with intellectual disability and a typically developing group matched on mental age. Pennington et al. [34] used a virtual version of the Morris water maze. This showed that individuals with DS had difficulty using landmarks within the environment to locate a hidden platform, i.e. they had poor configural knowledge. Edgin et al. [14] used the virtual Morris water maze used in Pennington et al. [34]; no differences were reported between a DS group and a mental-aged matched typically developing group aged 3 to 8 years. However, Edgin et al. [14] reported high rates of non-completion and error rates on this task so this result might not reflect true ability levels. As a precursor to the current study, Courbois et al. [9] investigated configural knowledge in a small group of participants with DS. Ten participants with DS learnt two short routes before being asked to find the shortest route from the beginning of one route to the end of the other. Performance was poor, with only two of the ten DS participants able to find the shortest route. This compared to a success rate of 50 % in a mental age-matched typically developing (TD) group and 100 % success rate in a chronological age-matched TD group and again suggests poor configural knowledge in DS.

In the current study, configural knowledge was assessed in addition to route knowledge in both DS and WS. Given that we know that route knowledge is available to both groups (e.g. [16–18, 36]), it is important to explore the deficit in configural knowledge in these groups. This was the largest investigation of large-scale configural knowledge to-date in both the DS and WS population, and the first time that cross-syndrome comparison of large-scale configural knowledge had been carried out.

**Fig. 1:**
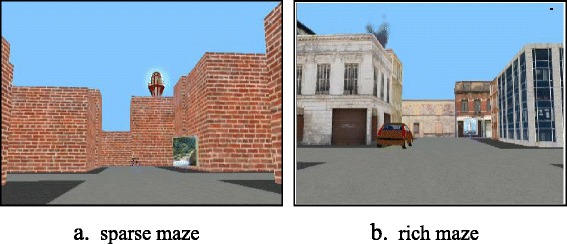
Screenshots of the sparse and rich maze. **a** Sparse maze. **b**. Rich maze

With the advent of easily accessible virtual reality, many studies now employ VEs to explore route learning ability. This has advantages over the real-world in terms of experimental control and aspects related to fatigue and the length of assessments. However, in controlling VEs to manipulate variables of interest such as junction type, the number and type of landmarks, and the layout of environments, most studies that have employed VEs have used relatively sparse environments, i.e. brick wall mazes and/or a limited set of landmarks. Although it has been shown that the skills used when navigating VEs map onto the same skills when navigating in the real world [40], it is important to determine how performance in sparse virtual environments compares to that in rich, more ecologically valid virtual environments. This was the second aim of the current study. Each participant took part in two conditions, one in which they learnt routes and were asked to find a shortcut in a sparse brick wall maze and one condition with an identical procedure, which employed a rich maze which featured buildings instead of brick walls (see Fig. 1). To our knowledge, comparison of performance in sparse vs. rich environments has hitherto not been made before either in typical or atypical groups.

We also investigated the possible differential contribution of different types of attention processes to route and configural knowledge. In a previous study, different components of attention differentiated the DS and WS groups from the typical groups with respect to route knowledge; response time on a Go/No Go task was related to performance in the TD group, whilst errors on the Go/No Go tasks predicted performance in the DS and WS groups [36]. This differentiation can be related to selective attention and inhibition, respectively. Indeed, difficulties with inhibition are consistent with previous research for both DS and WS [8, 28].

In the current study, we will determine the attentional mechanisms associated with route knowledge and with configural knowledge for sparse and for rich environments. Because attention, as measured by a single task, was predictive of route learning in Purser et al. [36] in a DS and WS group, we have included a larger battery of attention tasks here to gain a better understanding of this relationship. Given that distant landmarks are particularly useful cues when developing configural knowledge, and the requirement to switch from an egocentric to an allocentric representation of the environment, it is possible that selective attention and/or attention switching will be particularly important for the development of configural knowledge. We also predict that the attentional mechanisms required to navigate in a sparse or rich environment will differ on account of the stronger requirement to select appropriate information to attend to in the rich maze. The attention battery was chosen to reflect multi-component models of attention (e.g. [15, 30]) and thus measured: selective attention, sustained attention and attention switching. The Go/No Go task was also included as a measure of executive control and to provide a link between this study and Purser et al. [36].

## Methods

### Participants

There were three participant groups: 93 TD individuals (43 males, 50 females), 29 individuals with DS (20 males, 9 females) and 20 individuals with WS (10 males, 10 females). All DS and WS groups had received both phenotypic and genetic diagnosis by their clinician. The TD group was an opportunity sample from local schools, with no known diagnoses of neurodevelopmental disorders. Thirty-two of the TD group, 11 of the DS group and the whole WS group were English, and the remaining participants were French. Recruitment of all participants was via schools and parent-support groups. Raven’s Coloured Progressive Matrices (RCPM; [38]), a measure of non-verbal mental age, was administered to all participants. English participants with DS or WS were also tested on the British Picture Vocabulary Scale III (BPVS; [12]), a measure of receptive vocabulary, whereas the French participants with DS or WS were tested on the equivalent, L'échelle de vocabulaire en images Peabody (EVIP; [13]). For the sake of brevity, both tests will hereafter be referred to as BPVS. Comparison of group means demonstrated an effect of age (*F*(2, 139) = 187.28, *p* < .001, *η*_*p*_^2^ = .73). Tukey comparisons demonstrated that the TD group were younger than the two atypical groups (*p*s < .001), with no difference between the DS and WS groups (*p* = .12). There was a group difference for RCPM (*F*(2, 139) = 79.90, *p* < .001, *η*_*p*_^2^ = .54); the TD group was stronger than the DS and WS groups (*p* < .001), who showed no difference (*p* = .10). The WS group had higher BPVS scores than the DS group, *t* (47)=7.53, *p* < .001. Participant information (mean, standard deviation and range) is given in Table 1.

**Table 1:** Descriptive statistics for route learning and the cognitive test battery

	Mean	SD	Range
Age (years; months)			
TD	8;6	1;6	5;5–11;4
DS	19;11	4;0	14;5–26;5
WS	22;0	7;8	13;5–44;5
BPVS (raw)			
TD	-	-	-
DS	60	27	21–138
WS	117	25	70–159
RCPM (raw)			
TD	28	4.4	17–36
DS	17	4.0	10–27
WS	20	4.7	12–30
Route learning errors			
Rich			
TD	16	18	0–69
DS	53	58	1–252
WS	50	54	0–221
Sparse			
TD	14	18	0–97
DS	42	42	1–173
WS	43	37	3–126

*TD* typically developing, *DS* down syndrome, *WS* Williams syndrome, *BPVS* British picture vocabulary scale, *RCPM* Raven’s Coloured Progressive Matrices

### Maze task

The main experimental task was a VE maze, created using Virtools 5.0, a 3D software toolkit. The maze was laid out in a grid of 16 ‘blocks’. Fifteen landmarks featured on the paths in the maze.

There were two conditions: Rich and Sparse, each with the same layout and same number of landmarks. In the Sparse condition, the blocks were uniform brickwork and each appeared identical to every other (Fig. 1a). In the Rich condition, the blocks appeared as detailed and realistically rendered street blocks and each block was unique (Fig. 1b). In both conditions, there were three ‘sheds’ hidden in recesses within particular blocks. Each participant started the maze at a brown shed, which contained a locked box. There were two further sheds in each condition, a blue one and a green one, both of which contained a hidden key.

**Fig. 2:**
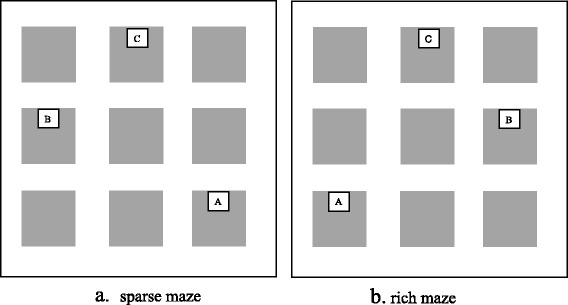
Layouts of the maze and treasure chests (*A*, *B* and *C*) for sparse and rich environments. **a** Sparse maze. **b** Rich maze

In each condition, there were two routes to learn: from the brown shed to the blue shed and back again (to open the box with the key) and from the brown shed to the green shed and back again (Fig. 2). To ensure equivalent difficulty levels across rich and sparse conditions, the routes in the sparse condition were mirror images of the routes in the rich condition.

There were two non-overlapping sets of landmarks, so that there were 2 × 2 possible combinations of conditions and landmark sets. Each participant completed one sparse condition and one rich condition; order of condition, order of route learned, and landmark set were fully counterbalanced across participants.

### Procedure

Participants took part in two sessions, spaced no more than 3 weeks apart. Each session started with one condition of the maze task (Rich or Sparse), followed either by the attention test battery (see below), or followed by RCPM (and also BPVS for the WS and DS groups). Maze task condition and the subsequent tasks were counterbalanced between the first and second sessions across participants.

Each participant first completed a short familiarisation maze with no landmarks and with plain blue walls. In the familiarisation maze, participants started next to a locked box of treasure and were instructed to go forward and find the key to open the box. Participants followed a single path which included making two right angle turns; there were no direction decisions to be made.

Movement through the maze was controlled by a combination of computer keyboard and mouse: the space bar caused forwards movement, whilst orientation was achieved by the mouse. Participants could orient upwards and downwards as well as orienting left and right. At the end of the maze was a key, which was picked up by walking into it. A key symbol appeared and remained at the edge of the screen to remind the participant that they were in possession of the key. On returning to the box and walking into it, there was an animation of the box opening to reveal a large golden star.

After the familiarisation maze, the participant was instructed that the main game was about to begin. It was explained again that the game involved finding the key to open the box of treasure, but that this time if the participant made a wrong turn, their path would be blocked by a red barrier, so they would need to try a different way. On an initial learning trial, all the barriers were visible, clearly blocking incorrect paths such that participants could only follow the correct route to the key and back. On subsequent trials, however, participants were instructed to walk the correct route from memory. Thus, barriers were invisible unless the participant attempted to pass down an incorrect path. Similarly, the barriers disappeared after the participant moved away from them. Each triggering of a barrier counted as an error. The participant repeated the entire return route, from box to key and back, until it was completed twice in a row without triggering any barrier, up to a maximum of ten attempts. The dependent variable was the number of errors made in total across all attempts at learning a route. If the participant travelled past the shed (with key hidden within) twice without noticing it, the experimenter said “I think you are close, stop and look around you”.

After this procedure for the first route in each condition, the participant was informed that a different route was about to be learned, in which the key was hidden in a different place from before. The procedure was then repeated for the second route. If a participant successfully learned both routes, they were tested on their ability to find a shortcut between the two key locations. If the participant learned the route to the blue shed first, the start point was the blue shed and the key would be hidden in the green shed. If the route to the green shed had been learned first, the start point was the green shed and the key would be hidden in the blue shed. The participant was instructed “You are going to start at the blue (or green) shed and need to go straight to the green (or blue) shed in the quickest way possible, get the key and come back to open the box. There are no barriers now, so you can go any way you want to. You need to go the quickest way, though. Once you have the key, go back the way you came and open the box.” The participant was given feedback as to whether the quickest route had been used each time. There was a time limit of 60 s for each attempt; there was a maximum of five attempts to find the quickest route on two consecutive trials; if successful, the participant was credited with knowledge of the shortcut.

### Attention test battery

#### Go/No Go task

Go/No Go (GNG) was chosen as a measure of executive control. A pseudo-random series of 5-cm diameter red, purple, orange and yellow solid circles was presented on a computer, against a white background. The participant was told that he/she was going to play the ‘White Game’ and was instructed to press the space bar as rapidly as possible when he/she saw each circle, unless it was red, in which case he/she should refrain from pressing the space bar. If the space bar was pressed on red, a buzzing ‘error’ noise was heard and the circle disappeared. Each circle disappeared after 2 s if the space bar was not pressed. If the participant pressed the space bar on two subsequent red trials, he/she was reminded of the task rules. Red trials constituted 1/4 of all trials. There were 8 practice trials, followed by 64 experimental trials. The dependent measures were the average reaction time for correct hits and the number of errors (pressing the space bar for a red circle).

#### Sustained attention task

A pseudo-random series of 5-cm diameter white, pink, green and blue solid circles was presented on a computer, against a black background. The participant was told that he/she was going to play the ‘Black Game’ and was instructed to press the space bar as rapidly as possible when he/she saw each blue circle, but to refrain from pressing the space bar on seeing all other colours. If the space bar was pressed on the other colours, or was not pressed on a blue trial, a buzzing ‘error’ noise was heard and the circle disappeared. Each circle disappeared after 2 s if the space bar was not pressed. If the participant failed to press the space bar on a blue trial, he/she was reminded of the task rules. Blue trials constituted 1/8 of all trials. There were 8 practice trials, followed by 64 experimental trials. The dependent measure was the average reaction time for correct hits.

#### Switching attention task

The participant was told that the Black and White Games would now be mixed together and was reminded of the rules for each. There were eight practice trials of the Black Game, followed by eight practice trials of the White Game. There then followed two 16-trial blocks of the Black Game, alternating with two 16-trial blocks of the White Game. The dependent measure was the average reaction time for correct hits in the White Game trials. A switching measure was calculated by subtracting this average from the average reaction time for correct hits in the main GNG task.

#### Selective attention task

The selective attention task was adapted from a ‘flanker’ task (e.g., [25]). Each stimulus consisted of a row of five arrows, with the target in the central position. Trials were either congruent, on which the arrows pointed in the same direction, <<<<< or >>>>>, or incongruent, >> < >> or << > <<. The participant’s task was to press the space bar as rapidly as possible only if the central arrow pointed left. The rules were explained with the aid of pointing to the left to clarify matters in case the participant was not confident with the left vs. right distinction. If the space bar was pressed when the central arrow pointed to the right, a buzzing ‘error’ noise was heard and the stimulus disappeared. Each trial’s stimulus disappeared after 1.5 s if the space bar was not pressed. If the participant failed to press the space bar on two consecutive ‘left’ trials, he/she was reminded of the task rules. ‘Left’ trials constituted 1/2 of all trials. There were 8 practice trials, followed by 64 experimental trials. The dependent measures were the average reaction time for correct hits on both congruent and incongruent ‘left’ trials. A selective attention measure was subsequently computed by subtracting the former from the latter.

## Results

### Route knowledge

#### Predictors of route knowledge

Each time that the participant hit a barrier, this counted as an error. The cumulative number of errors made across learning trials for the two routes was calculated for each participant. Correlations between overall route knowledge errors and other measures were calculated for each group separately (see Table 2 for descriptive statistics of the attention measures). There were extreme values on various measures in the DS and WS groups. However, there were no grounds for believing that these values reflected error rather than genuine cognitive variability. Therefore, these values were retained, but these groups’ data were analysed non-parametrically to avoid undue influence by individual data points. Correlations are shown in Table 3. These analyses were repeated for route knowledge errors in rich and sparse mazes separately (the sum of errors made across learning trials for the two learnt routes); there was little difference in the patterns of correlation, so these separate analyses have been omitted for brevity. The analysis was further repeated using z-scores (calculated from the mean and standard deviation of each group separately), as a way of normalising any differences in the extent of within-group variation across measures and across groups; this made no difference to the patterns of correlations and so we can be confident that the patterns of correlation observed in Table 3 are not related to reduced variability in any of the measures. As can be seen in Table 3, for the TD group, most measures significantly correlated with route knowledge errors. For the DS group, only RT in the switching attention Go/No Go task (this represents RT on the Go/No Go task when administered as an interleaved block with the sustained attention task; a combined measure of switching and executive control) predicted route knowledge. For the WS group, only RCPM predicted route knowledge.

**Table 2:** Descriptive statistics for the attention test battery

	Mean	SD	Range
Go/No Go task reaction time			
TD	0.58	0.14	0.38–1.16
DS	0.75	0.25	0.45–1.34
WS	0.61	0.13	0.35–0.83
Go/No Go errors			
TD	1.85	1.86	0–8
DS	1.97	2.15	0–8
WS	2.10	2.01	0–9
GNG RT in the switching condition			
TD	0.64	0.14	0.43–1.15
DS	0.84	1.89	0.53–1.31
WS	0.69	0.13	0.48–0.92
Switching reaction time			
TD	0.06	0.08	–0.15–0.28
DS	0.08	0.18	−0.30–0.41
WS	0.07	0.07	−0.08–0.22
Sustained attention reaction time			
TD	0.67	0.18	0.46–1.43
DS	0.81	0.24	0.51–1.42
WS	0.66	0.15	0.46–0.94
Selective attention reaction time			
TD	0.10	0.08	−0.07–0.42
DS	0.12	0.28	−0.39–0.91
WS	0.18	0.16	−0.07–0.53

*TD* typically developing, *DS* down syndrome, *WS* Williams syndrome

**Table 3:** Correlations between route learning errors and other measures

	Age	BPVS	RCPM	GNG RT	GNG error	SWGNG RT	SW RT	SU RT	SEL RT	Rich errors	Sparse errors
TD	−.43**	N/A	−.32**	.32**	.06	.28**	−.08	.36**	.24*	.80**	.66**
DS	−.02	−.22	−.22	.33	.13	.58**	.22	.26	−.18	.94**	.89**
WS	−.03	−.33	−.74**	.14	.13	−.06	−.23	.24	−.27	.91**	.90**

*BPVS* British picture vocabulary scale, *RCPM* Raven’s Coloured Progressive Matrices, *GNG RT* Go/No Go task reaction time, *GNG error* Go/No Go errors, *SWGNG RT* GNG RT in the switching condition, *SW RT* switching reaction time, *SU RT* sustained attention reaction time, *SEL RT* selective attention reaction time

*p < .05; **p < .01

#### Multiple regression

Only one significant predictor of route knowledge emerged from the correlational analysis for the DS and WS groups and so multiple regression would not be informative. To discover which variables uniquely predicted route knowledge performance in the TD group, three separate forwards stepwise multiple regression analyses were run, with overall errors, errors in the rich condition and errors in the sparse condition as dependent variables, and pure-blocks Go/No Go RT, switching-blocks Go/No Go RT, sustained attention RT and selective attention RT as predictors (i.e. the significant predictors from the correlational analyses). Age and RCPM were not included because they reflect a multitude of factors: the aim was to investigate the influence of cognitive components. The criterion for adding each variable was whether its addition caused a significant change in *F*. For overall route knowledge errors sustained, attention RT explained 8 % of variance, *F*-change (1,88) = 7.29, *p* = .008 and selective attention RT explained an additional 5 %, *F*-change (1,88) = 5.25, *p* = .025. For errors in the rich condition, sustained attention was the lone predictor, explaining 8 % of variance, *F*-change (1,88) = 8.00, *p* = .006. For errors in the sparse condition, selective attention RT alone explained 12 % of variance, *F*-change (1,88) = 11.56, *p* = .001.

#### Trajectory analyses

To compare developmental trajectories, parametric methods were necessary. Therefore, participants scoring more than 3 standard deviations above the group mean of the dependent variable (overall route knowledge errors) were excluded. Using this criterion, one participant was excluded from the WS group. In order for the ranges of the covariate to be similar across the groups, the four participants with the lowest RCPM scores in the DS group were also excluded. The ANCOVA model included interaction terms between the RCPM covariate and the between-subjects factor to explore whether route knowledge performance developed at a different rate in each group with reference to non-verbal ability. The data were analysed with respect to RCPM rather than chronological age because chronological age ranges were largely non-overlapping between the TD and disorder groups. So that any differences in the intercepts of the trajectories were meaningful, RCPM was rescaled such that the analysis reflected the intercept at the lowest RCPM score of the groups. This does not change the analysis, but aids interpretation.

Analyses of order effects were carried out prior to exploring differences across rich and sparse mazes. Route knowledge errors did not differ according to which landmark set was paired with which maze, *F* (1, 130) = 2.58, *p* = .15, *η*_*p*_^2^ = .02. There was, an interaction between maze order and maze type (*F* (1, 130) = 19.97, *p* < .001, *η*_*p*_^2^ = .13); participants performed better on the maze that they completed second, indicative of procedural practice effects (rich maze first, *t*(69) = 4.93, *p* < .001; sparse maze first, *t* (65) = −2.77, *p* = .007). This could not affect the pattern of results because counterbalancing of maze order neutralised the practice effect.

A mixed-design ANCOVA with route knowledge errors as the dependent variable, group as the between-subjects factor, maze type as the within-subjects factor and RCPM as the covariate was carried out. This revealed no main effect of maze type, *F* < 1, nor any reliable interaction of maze type and group, *F*(2130) = 1.37, *p* = .26, *η*_*p*_^2^ = .02, nor of maze type and RCPM, *F* < 1. The following analyses, therefore, reflect cumulative route knowledge errors across rich and sparse mazes.

Overall, route knowledge errors differed across groups, *F*(2, 133) = 20.35, *p* < .001, *η*_*p*_^2^ = .23, on account of fewer errors made by the TD group than the WS and DS groups (Sidak-adjusted post hoc tests, *p* < .05 for both), with no difference in errors between the two disordered groups (*p* > .05) (see Table 1 for mean route knowledge errors). A univariate ANCOVA with overall route knowledge errors as the dependent variable, group as the between-subjects factor and RCPM as the covariate revealed a significant group effect at the lowest level of non-verbal ability, *F*(2, 130) = 5.80, *p* = .004, *η*_*p*_^2^ = .082. Paired comparisons showed that the TD group made fewer errors than the WS group at the trajectory intercept, *F*(1, 107) = 26.04, *p* < .001, *η*_*p*_^2^ = .20, with no differences for other comparisons (DS vs. WS, *F*(1, 39) =1.65, *p* = .21, *η*_*p*_^2^ = .04; TD vs. DS, *F*(1, 114) = 2.43, *p* = .12, *η*_*p*_^2^ = .02). RCPM was reliably negatively related to route knowledge errors, *F*(1, 130) = 10.14, *p* = .002, *η*_*p*_^2^ = .072.

**Fig. 3:**
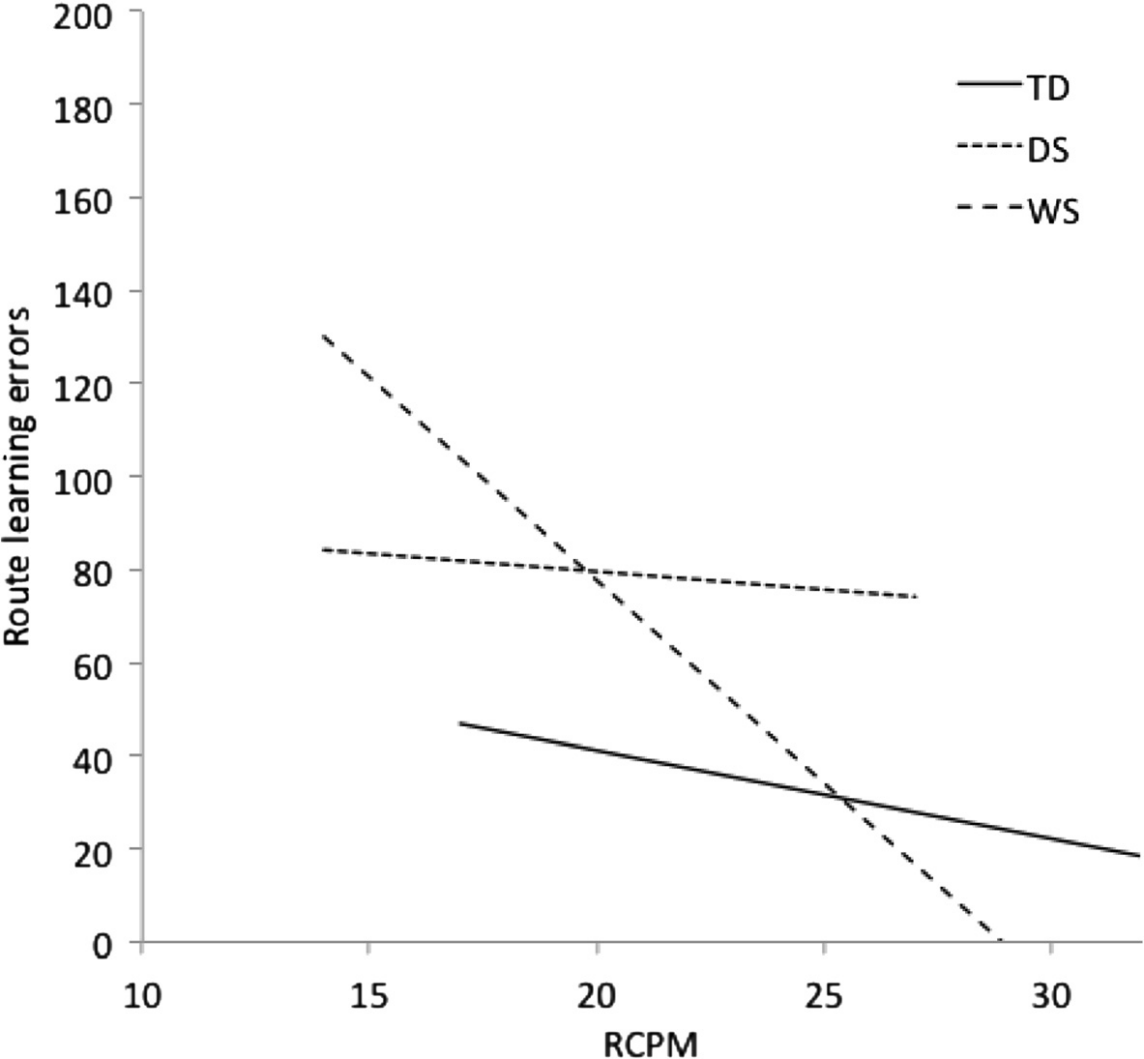
The relationship between overall route learning errors and RCPM score, by group. *TD* typically developing, *DS* Down syndrome, *WS* Williams syndrome, *RCPM* Raven’s Coloured Progressive Matrices

There was a significant interaction of group and RCPM (see Fig. 3), *F*(2, 130) = 4.27, *p* = .016, *η*_*p*_^2^ = .062, which is indicative of difference in the slopes of the developmental trajectories across groups. There was no reliable difference in trajectory slopes between the TD and DS groups, *F*(1, 114) < 1, nor the DS and WS groups, *F* (1,39) = 2.14, *p* = .152, *η*_*p*_^2^ = .052. However, the WS group’s route knowledge errors decreased more than the TD group’s with increasing RCPM score, *F* (1, 107) = 17.76, *p* < .001, *η*_*p*_^2^ = .142.

### Configural knowledge

#### Shortcut finding

**Fig. 4:**
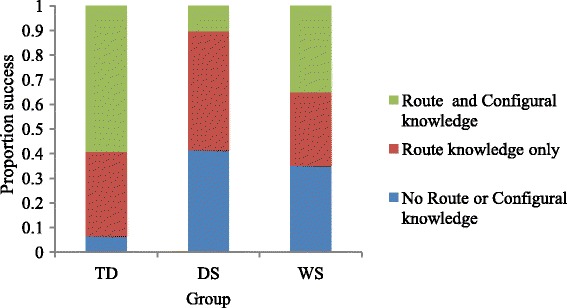
Success rate of gaining route knowledge and configural knowledge on at least one of the two mazes

Participants who successfully learnt the two routes to a criterion of two consecutive trials without error, i.e. those who had route knowledge, qualified for the shortcut assessment for that maze. Route knowledge was achieved in at least one of the two mazes for 87/93 (94 %) TD participants, 17/29 (59 %) DS participants and 13/20 (65 %) WS participants (see Fig. 4). Participants were credited with having found the shortcut if they succeeded in finding the shortcut on two consecutive trials in a maximum of five trials (two TD, one DS and two WS had learnt the two routes of a maze, but either refused to complete the shortcut assessment for that maze, or failed to understand the task requirements; these participants are credited with not finding the shortcut for that maze). Considering each group as a whole, 55/93 (59 %) TD participants successfully found the shortcut on at least one of the two mazes, whilst 3/29 (10 %) participants with DS found it, and 7/20 (35 %) of the WS group. Frequency data is shown in Fig. 4. Chi-squared analysis demonstrated a significant association between group and shortcut success, *χ*^2^ (2) = 22.29, *p* < .001. Adjusted standardised residuals demonstrated that this was due to an association with finding the shortcut for the TD group (adjusted standardised residuals ±4.4), with not finding the shortcut for the DS group (adjusted standardised residuals ±4.3) and no significant association for the WS group (adjusted standardised residuals ±1.0).

### Configural knowledge score

We observed that some participants walked a slightly longer ‘shortcut’, or walked the shortcut in one direction, but not for the return journey, thus showing evidence of configural knowledge without meeting the shortcut criterion. A configural knowledge score was created by scoring a participant’s routes for each shortcut trial. Specifically, each trial was scored from 0 to 6 as follows: using the shortcut (in both directions, score = 6; in a single direction, score = 4), using a longer ‘shortcut’ that included one additional block length (in both directions, score = 5; in a single direction, score = 3) or two additional block lengths (in both directions, score = 2; in a single direction, score =1). Random walking and walking along taught routes only received a score of 0. The sum of these scores over five trials provided a configural knowledge score out of 30 for each maze (if shortcut criterion was met before five trials, credit [a score of 6] was given for remaining trials), with a higher score reflecting stronger configural knowledge (maximum score, 30). Ten percent of the data was coded by a second coder and achieved high inter-rater reliability (Spearman’s *r* > .90 for both mazes). Configural knowledge scores are displayed in Table 4. The two TD, one DS and two WS participants who had route knowledge but did not complete the shortcut trials were not included in this analysis. Data was not normally distributed.

**Table 4:** Configural knowledge scores for each group on each maze

	Sparse maze			Rich maze		
	*N*	Median	IQR	*N*	Median	IQR
TD	79	17	14	77	22	21.50
DS	16	9	6.56	14	5.50	9.50
WS	11	16	21	10	20	22.88

*IQR* interquartile range

Kruskal-Wallis ANOVAs were carried out with a between-participant factor of group for each maze separately. This showed a significant effect of group for both mazes: sparse maze, *χ*^2^(2, *N* = 106) = 6.76, *p* = .03; rich maze, *χ*^2^(2, *N* = 101) = 12.47, *p* = .002. For both mazes, this was accounted for by poor performance in the DS group. That is, in the sparse maze, the DS group were poorer than the TD group only (TD vs. DS, *Z* = −2.53, *N*_*A*_ = 79, *N*_*B*_ = 16, *p* = .01; TD vs. WS, *Z* = −1.01, *N*_*A*_ = 79, *N*_*B*_ = 11, *p* = .32; DS vs. WS, *Z* = −0.74, *N*_*A*_ = 16, *N*_*B*_ = 11, *p* = .48), whilst in the rich maze, the DS group were poorer than the TD group (*Z* = −3.52, *N*_*A*_ = 77, *N*_*B*_ = 14, *p* < .001) and the WS group (*Z* = −2.06, *N*_*A*_ = 14, *N*_*B*_ = 10, *p* = .04) (TD vs. WS, *Z* = −0.52, *N*_*A*_ = 77, *N*_*B*_ = 10, *p* = .60).

Comparison across Rich and Sparse mazes is of limited value for the disorder groups given the low group numbers who qualified for the shortcut trials for both mazes. Nonetheless, analyses for each of the three groups using Wilcoxon signed ranks tests showed no advantage in configural knowledge score for one maze over the other: TD, *Z* = −1.15, *N* = 69, *p* = .25; DS, *Z* = −.75, *N* = 13, *p* = .46; WS, *Z* = −.95, *N* = 8, *p* = .40.

Spearman correlations for each group between configural knowledge score and the predictor variables demonstrated a correlation between the rich maze configural knowledge score and switching-blocks Go/No Go Error for the WS group *r* = −.80, *p* = .005, *N* = 10, and between sparse configural knowledge score and RCPM (*r* = .232, *p* = .032, *N* = 79) for the TD group; with no significant correlations for the DS group.

#### Walked distance

To determine learning across trials, the distance walked in the shortcut trials was calculated for the rich and the sparse maze (with the exception of the two TD, one DS and two WS participants who did not provide data, mentioned above) (see Table 5). Learning was assessed by comparing the walked distances on the first and last shortcut trials. Due to some positive skewness in the data, non-parametric analyses were conducted.

**Table 5:** Walked distance for each group on first and final shortcut trials for each maze

	Sparse maze					Rich maze				
	*N*	First trial		Final trial		*N*	First trial		Final trial	
		Median	IQR	Median	IQR		Median	IQR	Median	IQR
TD	77	650.59	315.05	375.65	248.24	77	539.09	344.51	342.62	352.71
DS	16	511.74	283.98	611.90	368.33	13	504.47	316.56	460.40	367.05
WS	10	530.08	133.82	431.26	252.18	8	529.08	314.45	430.08	451.24

*IQR* interquartile range

In both mazes, the TD group had shorter walked distances in the last compared to the first trial, sparse maze, *Z* = −5.63, *N* = 77, *p* < .001; rich maze, *Z* = −3.35, *N* = 77, *p* = .001, whilst the disordered groups showed no difference between the first and last trials; sparse maze, DS, *Z* = −0.98, *N* = 16, *p* = .33; sparse maze, WS, *Z* = −0.65, *N* = 10, *p* = .52; rich maze, DS, *Z* = −0.59, *N* = 13, *p* = .55; rich maze, WS, *Z* = −0.84, *N* = 8, *p* = .40.

To determine whether learning in the TD group differed significantly from the atypical groups, signed difference scores were created by subtracting the walked distance on the last trial from that of the first trial. Kruskal-Wallis ANOVA revealed a significant group difference in learning for the sparse maze only (sparse, *χ*^2^ (2, *N* = 103) = 13.58, *p* = .001; rich, *χ*^2^ (2, *N* = 98) = 4.90, *p* = .086). This group difference was due to stronger learning in the TD group than the DS group: TD vs. DS, *Z* = −3.38, *N*_*A*_ = 77, *N*_*B*_ = 16, *p* = .001 and marginally stronger learning than the WS group: TD vs. WS, *Z* = −1.85, *N*_*A*_ = 77, *N*_*B*_ = 10, *p* = .064 (DS vs. WS, *Z* = −1.27, *N*_*A*_ = 16, *N*_*B*_ = 10, *p* = .21).

The same set of analyses was run again, excluding participants who succeeded in meeting the criteria for having found the shortcut for that maze. This was to characterise any learning that took place even if the shortcut was not found. For the TD group, the difference between first and last walked distances only remained for the sparse maze (sparse maze, *Z* = −2.77, *N* = 41, *p* = .006; rich maze, *Z* = −0.77, *N* = 35, *p* = .44). For the DS and WS groups, there was no evidence of learning across trials, DS sparse maze, *Z* = −1.60, *N* = 14, *p* = .11; DS rich maze, *Z* = −1.02, *N* = 12, *p* = .31; WS sparse maze, *Z* = −1.83, *N* = 5, *p* = .068; WS rich maze, *Z* = −1.83, *N* = 4, *p* = .068. Group differences in the effect of learning remained significant for the sparse maze, on account of stronger learning in the TD than DS group only, *χ*^2^ (2, *N* = 60) = 8.36, *p* = .02 (TD vs. DS, *p* = .006; TD vs. WS, *p* = .17; DS vs. WS, *p* = .52) and was non-significant for the rich maze, *χ*^2^ (2, *N* = 51) = 3.04, *p* = .22.

#### Alternative strategies

Some participants were observed using an alternative strategy which involved summing the two taught routes in order to get from the green to the blue shed (or vice versa). Arguably, this does not require configural knowledge at all, but relies on route knowledge instead. A taught route strategy was classified as walking the summed length of the two taught routes either in one direction or both directions on a shortcut trial. A second coder coded 10 % of the data with 100 % agreement across raters. Only a handful of participants used this strategy more than once. Thus, we analysed whether participants used a taught route strategy during any of their shortcut trials (a maximum of ten trials [five sparse maze, five rich maze trials]), but recognise that this does not necessarily reflect consistent use of this strategy. Chi-squared analysis demonstrated a significant association between using a taught route strategy and group, *χ*^2^ (3) = 9.34, *p* = .01. The DS group used a taught route strategy on at least one occasion significantly more frequently (7 out of 18 DS participants; adjusted standardised residuals ±2.6)) than the TD group (29 out of 88 participants; adjusted standardised residuals ±0.7), whilst the WS group used this strategy significantly less than the TD group (1 out of 12 WS participants; adjusted standardised residuals ±2.0).

## Discussion

In a field in which virtual environments are being increasingly used to investigate navigation skills, our finding that the use of sparse vs. rich environments has little impact on performance is of crucial importance to the design of future studies. Whilst our rich environment appeared more ecologically valid, this did not present an advantage (or disadvantage) for typically developing children or individuals with DS or WS. This finding validates the results of studies that have been conducted with relatively sparse environments as an accurate reflection of navigation performance and compliments knowledge that performance in VEs reflects performance in the real world [40].

This was also the first study to fully explore large-scale configural knowledge as a cross-syndrome comparison of DS and WS. It is evident that configural knowledge is problematic for these groups. For those participants with DS and WS who demonstrated route knowledge of the environment, a large proportion were unable to develop their spatial understanding beyond route knowledge to gain knowledge of the configural structure of the environment. Furthermore, in their endeavour to find the shortcut, neither group demonstrated any improvement from their first attempt to their final attempt. This suggests that the lack of large-scale configural knowledge would not have been ameliorated had we provided additional practice or experience in the VE.

This study also presented previously undemonstrated complexities in the ability to develop route knowledge. In the current study, where the environments were grid structures and participants were instructed to learn routes to a goal and back, individuals with DS and WS showed substantial impairments. That is, just under half of participants failed to gain route knowledge within ten learning trials. Given that route knowledge can be gained by these groups (e.g. [17, 18, 36]), it is possible that the difficulties demonstrated in the current study could be ameliorated through extensive repetition (beyond ten trials). Configural knowledge and route knowledge and the comparison between sparse and rich environments are discussed in detail below.

Route knowledge was associated with performance on the attention measures in the TD group (predominantly sustained attention and selective attention). This finding, discussed in detail below, highlights the importance of attentional mechanisms as processes which contribute to the development of navigation skills. Configural knowledge was related to RCPM score only, highlighting the strong non-verbal/visuo-spatial component of this sophisticated navigational ability. For the DS and WS groups, only the combined measure of attention switching and executive control was related to performance (it was related to route knowledge for the DS group and configural knowledge for the WS group). This suggests that the known weaknesses in attention [3] in these groups do not cause substantial limitations to navigation for these group, or that other factors are substantially more limiting, such as to overshadow any negative impact of impaired attentional mechanisms on performance. The relationship between non-verbal ability and route knowledge in the WS group supports this notion.

### Route knowledge

The routes in the current VEs differed from those that have been used to-date with these groups. The current design involved learning a route through a grid maze of cross-junctions which are arguable, the hardest kind of junction to feature on a route because each decision point featured a choice between three potential paths. In addition, participants’ route knowledge in this study was a measure of their ability to learn the route from a starting position to a goal and back again to the start, hereafter referred to as a return route. These features represented a substantial challenge; just under half of participants with DS and WS failed to gain route knowledge of both routes within a particular maze in the current study. This compared to success from almost all DS and WS participants and young TD children on a single direction (i.e. from a start position to a goal only) T- and L-junction designs [17, 18, 36].

We employed a more expansive battery of attention measures than used in previous studies. As expected, this demonstrated differential associations with route knowledge errors across groups. The DS group showed a relationship between route knowledge errors and RT on the switching blocks of the Go/No Go task, a combined measure of attention switching and executive control, whilst for the WS group, route knowledge errors were only related to RCPM score, a measure of non-verbal cognition. For the TD group, whilst almost all attention, age and IQ measures correlated with route knowledge errors, of the attention measures, sustained attention RT was the best predictor, with some input from selective attention RT. Chronological age was not related to route knowledge in the DS or WS group. This is not surprising as it is widely recognised that level of cognitive impairment is rarely related to age in these groups [23].

The current study shows consistency with Purser et al. [36] for the TD group, where route knowledge was related to Go/No Go RT. Although the Go/No Go task is predominantly a measure of executive control, reaction time is an index of the general attentional resources required to attend to the task and might reflect sustained attention [39]. Thus, despite the more challenging task demands of the current study, there is overlap in the mechanisms employed to complete the task by the typical population. The association with sustained and selective attention in the current study is logical. That is, route learning involves selecting the appropriate subset of landmarks to encode. For route knowledge, this enables useful landmarks to be paired with junctions, with reduced attention to less useful landmarks, thus facilitating decisions at junctions. Sustaining attention along the full length of the route enables a person to build knowledge of the entire route. Sustained attention was the stronger predictor in the rich environment and selective attention in the sparse environment. As discussed later, this likely reflects a stronger need to maintain attention on the task at hand in the rich maze due to the amount of potentially distracting information available.

For the atypical groups, the current results demonstrate that impaired route knowledge in DS or WS is not an artefact of having learning difficulties; the differential association between route knowledge and attention switching and executive control for the DS group but non-verbal ability for the WS group reflect syndrome-specific deficits. This pattern, however, shows some discrepancy from Purser et al. [36] in which route knowledge errors were associated with Go/No Go errors (inhibition), as well as RCPM performance (non-verbal cognition), for *both* groups. This difference might reflect the differing task demands of the two studies coupled with the syndrome-specific profiles of strengths and weaknesses. The association between route knowledge and attention switching and executive control in the DS group could be specific to the kind of route knowledge measured here, i.e. learning return routes. We suggest that the act of changing direction in a return route requires attentional switching given that the order of turns and landmarks is reversed in one direction compared to the other. As attention switching is poor in DS [3, 7], this could have acted as a limiting factor in acquiring knowledge of return routes. Training this skill, therefore, might have ameliorating effects on the ability to learn such complex routes. However, this does not marry well with impaired attention switching in WS [3], yet the lack of association between attention switching and route knowledge for this group. We tentatively argue that the differences between the two atypical groups, therefore, reflect the syndromic characteristic of poor non-verbal cognition in WS. RCPM performance was associated with route knowledge in the WS group and thus appears to represent a limiting factor when acquiring route knowledge. The demands on perspective taking and mental rotation, i.e., non-verbal cognition, are stronger for return routes as used here, than single direction routes, as used in previous studies. Within the profile of non-verbal cognition in WS, mental rotation and perspective taking performance represent weak areas of ability (e.g. [1]); the additional demands on these mechanisms might explain why return route knowledge was solely related to RCPM in the current study, over and above any input from impaired attention mechanisms.

Developmental trajectory analysis of route knowledge was carried out using RCPM score as a measure of non-verbal mental age. The developmental trajectories showed an atypical developmental trajectory for the WS group; significantly more errors than the TD group were made at low levels of non-verbal ability, but the steeper rate of development in the WS group enabled them to catch-up with TD route knowledge performance at higher levels of non-verbal ability. This suggests that the strong association with non-verbal ability is particularly limiting to route knowledge at low levels of non-verbal ability. That is, at higher levels of non-verbal ability, this group perform at the level expected for their non-verbal mental age. This has implications for intervention in WS. That is, training in non-verbal skills could positively impact navigation performance. However, given that the RCPM reflects a multitude of non-verbal skills, further research would be required to determine the precise nature of non-verbal training that would have the best chance of being effective. Based on previous evidence, likely candidates would be mental rotation and perspective taking [1] (although, note that impaired non-verbal cognition is a central characteristic of WS and thus the potential for improvement is limited).

Developmental trajectories for the DS group demonstrated that they consistently made significantly more errors than the TD group regardless of their level of non-verbal ability. This indicates that their route knowledge deficit remains constant across development and is likely impervious to intervention which concentrates on non-verbal abilities. The pattern here contrasts to the ‘catching-up’ trajectory observed in DS for single-direction route with L- and T-junctions [36], thus suggesting that route knowledge, at least in its simplest form, can show some improvement with increased non-verbal ability in DS.

### Configural knowledge

Both individuals with DS and individuals with WS demonstrated a deficit in configural knowledge. However, this cannot be accounted for simply by having learning difficulties. This is because each group showed different levels and patterns of performance. First, the impairment was more evident in DS than in WS; only 10 % of DS participants found the shortcut, compared to 35 % of the WS group; and differences in configural knowledge scores across groups were due to poor performance in the DS group. WS configural knowledge score did not reflect as much shortcut success as the TD group, but it was not statistically different from that of the TD group (although note that a lower percentage of individuals with WS [65 %] were offered the shortcut trials than the TD group [94 %]). Second, the DS group was more likely than the WS or TD groups to employ a compensatory strategy in which they simply walked the two taught routes end to end to get from the start to the finish. This does not require any configural information at all and highlights their reliance on route knowledge to find their way. Thus, although the DS group found it difficult to learn a return route, once a route had been learnt, they were able to rely on it.

A deficit in configural knowledge in DS is consistent with previous research which has demonstrated poor configural knowledge in DS [9, 34]. Specifically, Courbois et al. [9] also reported low rates of shortcut success (two of their ten participants found the shortcut), and a similar use of tagging together the two short routes as an alternative strategy to achieve the goal of getting from the start to the end. Configural knowledge during navigation has only hitherto been assessed in two studies with individuals with WS. Broadbent et al. and Farran et al. [16] demonstrated a deficit in configural knowledge in WS, in virtual and real-world environments, respectively. The current results are consistent with this; the majority of individuals with WS were unable to develop configural knowledge in an environment with which they had become familiar. This further demonstrates, in line with Broadbent et al., that the impairments observed in Farran et al. [16] reflect real deficit rather than a lack of familiarity with the environment. The current results also tally with reports of impaired configural knowledge in large-scale visual search and re-orientation tasks and in small-scale tasks [21, 26, 27, 33, 43]. The TD data mirrors Bullen et al. (2010) and Broadbent et al. [4] who demonstrated an increase in configural knowledge score with increasing age for a similar age range to that used here.

Participants in the current study were asked to determine return route shortcuts. Given that return routes in route knowledge increased the failure rate, relative to previous studies, we cannot rule out that this also impacted configural knowledge. If we had asked for a single direction route, success rates might have been higher. However, this is unlikely; first, there was no evidence of learning in the DS and WS groups; second, configural knowledge scores did not reflect shortcut success in one direction.

We were also able to access the learning process. TD participants demonstrated shorter walked routes on their last attempt to find the shortcut compared to their first. For the rich maze, this was accounted for by those TD children who found the shortcut, but for the sparse maze, learning was evident even in those who did not find the shortcut. This suggests that some TD participants were able to gain additional knowledge of the spatial relations between parts of the environment whilst attempting to find the shortcut, even if they did not meet the criterion for finding the shortcut. It is possible that reductions in walked distance resulted from learning using alternative strategies such as a route-based strategy, but this would rely on the ability to initially determine the route, which itself relies on configural understanding. Taken together, the reduction in walked routes from the first to the last attempt points to the benefits of repeated exposure to an environment in developing configural knowledge for TD children, with the caveat that this appears to be characteristic of sparse, less busy and distracting environments only. In contrast, the DS and WS groups showed no evidence of learning; as discussed above, this suggests that repetition has limited potential to facilitate the configural knowledge of these groups.

It was not possible to determine the mechanisms used to drive configural knowledge in the DS group. This is likely because too few DS participants demonstrated configural knowledge for associations to be detected. For the WS group, the combined switching/executive control measure was associated with configural knowledge on the rich maze. As noted above, this measure was related to route knowledge for the DS group. Perhaps, as before, this reflects switching between perspectives. For configural knowledge, this holds a different emphasis as the participant is not switching perspectives to simply reverse a route, but to determine the relationships across spatial locations in the VE. The switching component might also reflect the ability to switch between spatial frames of reference, for example an egocentric representation or first person perspective of the current view, vs. allocentric knowledge of the configural structure of the environment. This is supported by Broadbent et al. [1] who demonstrated impaired perspective taking in WS and thus tentatively raises the possibility that training perspective taking skills could foster the development of configural knowledge in WS. For the TD group, non-verbal ability was related to configural knowledge score on the sparse maze. This might reflect a reliance on complex spatial competencies such as employing a mental representation of the environment, a cognitive map. This results contrasts to the reliance on attentional measures for the TD group when gaining route knowledge, thus demonstrating the step change in the non-verbal cognitive processing required to develop configural knowledge, relative to route knowledge. The associations discussed were not consistent across mazes, which suggest that the mazes had slightly differing task demands, discussed below.

### Sparse vs. rich environments

The majority of studies that have used VEs have employed relatively sparse environments. In this study, we contrasted sparse and rich environments for the first time. Interestingly, and of importance to the design of future studies, this had little impact on performance. The ability to learn the two taught routes was comparable across the two maze types for all groups. The patterns of correlations with route knowledge were broadly similar, with the exception of subtle differences in associations for the TD group. That is, there were stronger associations with selective attention for the sparse maze and with sustained attention for the rich maze. The variance explained by these attention measures left a lot of variance unexplained and so they are by no means the sole or dominant contributor to route knowledge. Despite this, the associations are reasonable given the visual differences between the two maze types. In the sparse maze, the landmarks stood out from the background of brick walls, and thus the association with selective attention likely reflects TD participants’ efforts to attend to the most useful subset of landmarks as an aid to learning the route. In contrast, in the rich maze, the background consisted of buildings. This richness had the potential to distract TD participants from the task at hand of learning the route. Thus, those participants who had strong sustained attention (i.e. were able to stay on task) had stronger route knowledge. Configural knowledge scores and walked distances were also broadly similar across maze types. Evidence of learning in the TD group was stronger for the sparse maze than the rich maze (i.e. learning was evident even in those who did not meet criterion for finding the shortcut), and correlations with non-verbal ability was evident for the sparse but not the rich maze. This suggests that the more varied information in the rich maze facilitated more mixed strategies for route learning, supported by different mechanisms that were not revealed by whole sample analyses. Overall, with reference to absolute performance, it appears that richer information is no more distracting or engaging, than the sparse visual array presented in sparse VEs, and that studies which have used sparse environments are tapping into broadly the same processes as used in the arguably more ecologically valid rich environments. One caveat to this conclusion is that, although the use of buildings as opposed to brick walls made the rich VE richer than the sparse VE, both environments had the same number of landmarks. It is possible that an environment which is made richer by including a higher number of landmarks might have impacted performance to a greater extent than did the rich VE employed here.

## Conclusions

In conclusion, we have demonstrated impaired configural knowledge in two groups with neurodevelopmental disorders. The deficit was stronger in DS than in WS to the extent that the DS group often employed an alternative strategy of tagging one taught route onto another to reach a goal. This configural knowledge impairment has implications for independence for these groups, as route learning strategies that rely on route knowledge alone are inflexible to change (e.g. change to a learnt route due to a route being blocked by roadworks, or a detoured bus route), and it is difficult to get back on track when lost, without access to some configural understanding of the environment. We have also further characterised the development of configural knowledge in TD children and for the first time underlined the predominant mechanisms which drive the development of configural knowledge (non-verbal ability).

Furthermore, we have explored route knowledge for relatively complex routes which involved remembering a fixed route in both directions (a return route). This additional demand, relative to single-direction routes, coupled with the use of a grid-maze proved difficult for the DS and WS groups, who often did not gain route knowledge. This has implications for previously drawn conclusions that route learning is slow, but unimpaired in these groups. The additional demands drew on mechanisms that were themselves impaired in these groups (attention switching/executive control in DS and non-verbal cognition in WS), thus limiting their ability to learn the routes. These relationships provide some suggestions for training. That is, training individuals with WS or DS in skills such as attention switching (DS), non-verbal ability (WS) and executive control (WS) could positively impact their route learning abilities. Furthermore, verbal prompts regarding the relationships between landmarks and the spatial location of landmarks (e.g. [16]) could act to support attentional processes in these groups, with a downstream effect on route learning. Given that in the real world, it is usually important that an individual can not only find their way somewhere but also find their way back, the limitations of individuals with DS and individuals with WS in the current study have serious negative implications for these groups’ ability to navigate in the real world.
